# Efficacy of entomopathogenic nematodes at different spray pressures on *Stomoxys calcitrans* larvae (Diptera: Muscidae) in by-products of sugarcane mills

**DOI:** 10.1590/S1984-29612025059

**Published:** 2025-10-20

**Authors:** Américo de Castro Monteiro, Danielle Pereira da Silva, Gabriela Pereira Salça de Almeida, Vinícius Teixeira de Souza, Melissa Carvalho Machado do Couto Chambarelli, João Luiz Lopes Monteiro, Avelino José Bittencourt

**Affiliations:** 1 Universidade Federal Rural do Rio de Janeiro – UFRRJ, Instituto de Veterinária, Programa de Pós-Graduação em Ciências Veterinárias, Departamento de Parasitologia Animal, Seropédica, RJ, Brasil; 2 Universidade Federal de Roraima – UFRR, Centro de Ciências Agrárias, Departamento de Fitotecnia, Boa Vista, RR, Brasil; 3 Universidade Federal Rural do Rio de Janeiro – UFRRJ, Instituto de Veterinária, Programa de Pós-Graduação em Ciências Veterinárias, Departamento de Medicina e Cirurgia Veterinária, Seropédica, RJ, Brasil

**Keywords:** Biological control, stable fly, Heterorhabditis, spraying, sugarcane, Controle biológico, mosca-dos-estábulos, Heterorhabditis, pulverização, cana-de-açúcar

## Abstract

*Stomoxys calcitrans* is a hematophagous dipteran. The study aimed to evaluate the influence of spraying pressure on the efficacy of entomopathogenic nematodes (EPNs) on *S. calcitrans* larvae in sugarcane byproducts. Infectious juveniles (IJs) of *Heterorhabditis bacteriophora*, *Heterorhabditis baujardi* and *Heterorhabditis indica* were applied (200 IJs/larva) in water or 50% vinasse suspensions at pressures of 60, 70 and 80 psi to sugarcane straw, sugarcane bagasse and filter cake in plastic trays containing larvae. Control groups were not subjected to spraying. The efficacy of EPNs carried in water was not significantly reduced by spraying pressure when applied to bagasse and filter cake, but a lower larvae mortality was observed in straw for all EPNs applied at 80 psi and for *H. baujardi* and *H. indica* at 60 and 70 psi. Spraying of EPNs in vinasse did not significantly reduce the efficacy of all species in bagasse, but a reduction was observed in straw and filter cake depending on pressure. EPNs caused above 80% larvae mortality in most experiments. Spray-applied EPNs can infect and kill stable fly larvae in sugarcane substrates; however, their efficacy is influenced by spray pressure and carrier, as well as the treated substrate itself.

## Resumo

*Stomoxys calcitrans* é um díptero hematófago. O estudo objetivou avaliar a influência da pressão de pulverização na eficácia de nematódeos entomopatogênicos (NEPs) sobre larvas de *S. calcitrans* em subprodutos da cana-de-açúcar. Juvenis infectantes (JIs) de *Heterorhabditis bacteriophora*, *Heterorhabditis baujardi* e *Heterorhabditis indica* foram aplicados (200 JIs/larva) em água ou suspensões de vinhaça a 50% nas pressões de 60, 70 e 80 psi em palha de cana, bagaço e torta de filtro em recipientes plásticos contendo larvas. Os grupos controle não foram submetidos à pulverização. A eficácia dos NEPs em água não foi significativamente reduzida pela pressão de pulverização quando aplicados ao bagaço e torta, mas uma menor mortalidade larvar foi observada na palha para todos os NEPs aplicados a 80 psi e para *H. baujardi* e *H. indica* a 60 e 70 psi. A pulverização de NEPs em vinhaça não reduziu significativamente a eficácia das espécies no bagaço, mas observou-se uma redução na palha e torta dependendo da pressão. NEPs causaram mortalidade acima de 80% na maioria dos experimentos. NEPs aplicados por pulverização podem infectar e matar larvas da mosca em substratos de cana; porém, sua eficácia é influenciada pela pressão de pulverização e pelo veículo, bem como pelo próprio substrato tratado.

## Introduction

*Stomoxys calcitrans* Linnaeus, 1758 is a hematophagous dipteran known as “stable fly”, capable of parasitizing several animal species, including cattle, horses, sheep, goats, pigs, dogs, and cats. It can also feed on wild animals, birds, and even humans (Bittencourt, 2012). The stable fly has a cosmopolitan distribution, with a population increase in the hottest periods of the year (Barros et al., 2010). The seasonality of *S. calcitrans* shows two annual peaks in tropical and subtropical countries, which are related to warmer and wetter periods, although the fly is present throughout the year (Mullens & Meyer, 1987). Dominghetti et al. (2015) reported a higher abundance of the insect in the Brazilian Midwest between April/May and December. This coincides with the period of sugarcane harvest when the temperatures range between 18.6 and 26.8ºC.

This fly leads to considerable economic losses, with estimates of US $2.221 billion (Taylor et al., 2012) in the United States, and US $335.5 million per year in Brazil (Grisi et al., 2014). It is important to note that these values are underestimated, as they do not account for the recent outbreaks of this insect, especially in the Midwest and Southeast regions of Brazil (Souza et al., 2021).

The outbreaks of *S. calcitrans* in Brazil are closely related to the expansion of the sugarcane agroindustry. This is because the fertigation of sugarcane crops with vinasse and other sugarcane by-products helps maintain moisture and favors the fermentation of organic matter deposited on the soil. This activity also attracts and stimulates egg-laying by stable flies (Corrêa et al., 2013) and the large amount of organic matter makes the environment suitable for the development of their immatures. Adult flies emerge from these by-products and fly to neighboring properties, attacking animals and even humans (Souza et al., 2021).

The frequent and inadequate use of chemical insecticides for parasitic control has reduced the efficacy of these substances, particularly those belonging to the pyrethroid class (Barros et al., 2019). As a result, new control methods for this livestock pest are needed. In this context, biological control becomes a valuable alternative, minimizing the accumulation of chemical residues in agricultural products and reducing the resistance of pests to chemical compounds (Alves, 1998). Several agents can be used in biological pest control, including fungi, bacteria, viruses, and entomopathogenic nematodes (EPNs).

There are several reports in the literature demonstrating the potential of EPNs for controlling various agricultural pests, particularly those that have at least one developmental stage in the soil (Dolinski & Moino, 2006). Industrial-scale nematode production is already being carried out by companies in the USA, Europe, Cuba, Japan, and Israel. In Brazil, three EPNs-based products are already registered. Recently, Koopert do Brasil Holding S.A. began marketing a product based on EPNs from the species *Steinernema carpocapsae* Weiser, 1955 indicated for use in controlling agricultural pests. Among these pests is the sugarcane weevil, *Sphenophorus levis* Laurie, 1978 (Coleoptera Curculionidae). This opens up a great possibility for the use of EPNs in sugarcane fields to control stable flies, as the use of these organisms in sugarcane crops is not new.

The low cost of large-scale production of these agents (in arthropod hosts or artificial systems), the ability of EPNs to withstand long storage periods (Taylor et al., 1998), compatibility and ease of application in the field via irrigation/spraying systems, compatibility with most chemical pesticides, harmlessness to other invertebrates and vertebrates, and the high specificity of nematode lineages, thus preventing indiscriminate arthropod mortality and avoiding undesirable effects on the environment (Koppenhöfer & Grewal, 2005), make EPNs potential organisms for controlling pests in agriculture and livestock, especially those that have at least one developmental stage in the soil (Dolinski, 2006).

In Brazil, the use of EPNs has been studied mainly aiming at the control of arthropod pests of agriculture, presenting high mortality rates. In the last decade, Monteiro et al. (2014) took an important step towards the control of pests that affect domestic animals, using EPNs to control the tick *Rhipicephalus (Boophilus) microplus* Canestrini, 1888 in an insect-cadaver formulation (*Galleria mellonella* Hubner, 1813).

Monteiro-Sobrinho et al. (2016) and Leal et al. (2017) initiated, in Brazil, studies on the control of *S. calcitrans* using entomopathogenic nematodes, observing promising results in the use of EPNs on immature stages of the stable fly.

The present study aimed to evaluate *in vitro* the influence of spraying pressure on the viability and infectivity of EPNs, carried in water and 50% vinasse, to *S. calcitrans* larvae in by-products of the ethanol industry.

## Materials and Methods

### Insect

The *S. calcitrans* colony used in this study was established on a bench in the laboratory environment (27±1 °C and 70-80% relative humidity – RH), the colony originated from wild flies captured according to the method described by Mello (1989) and Moraes (2007) with adaptation.

### Entomopathogenic nematodes

The EPN colony was created using the method outlined by Lindegren et al. (1993), and it was maintained through *in vivo* multiplication in *Galleria mellonella* (Lepidoptera: Pyralidae). Infective juveniles (IJs) were held in a climate chamber (Eletrolab®, model EL 202/4) at 16 ± 1 °C and 70-80% RH in a 40 mL cell culture flask. IJs were counted in twelve 10 μL aliquots taken from aqueous EPN suspension to calculate the doses used in this study. After counting the IJs in the 12 aliquots, the highest and lowest number of EPN/aliquot were discarded and the average number of IJs in the remaining 10 aliquots was calculated. Based on this calculation, the concentration of the suspensions was adjusted to IJs/mL (Taylor et al., 1998). The nematodes used in the experiments were captured directly from White's traps (White, 1927) and used immediately after collection. The species used were *Heterorhabditis bacteriophora* HP88 Poinar, 1976, *H. baujardi* LPP7 Phan, Subbotin, Nguyen & Moens, 2003 and *H. indica* LPP30 Poinar, Karunakar & David, 1992.

### Pressurization system and collection of EPNs

**Figure 1:**
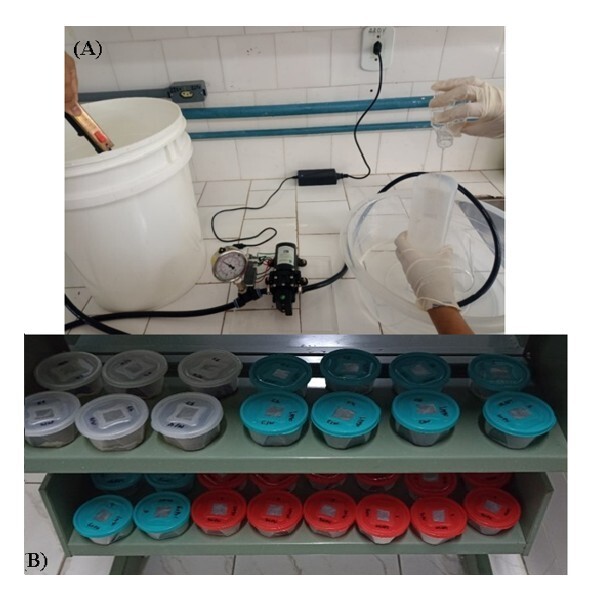
(A) Passage of entomopathogenic nematodes through the pressure system. (B) Biological assay to evaluate the mortality of *Stomoxys calcitrans* larvae caused by entomopathogenic nematodes.

The pressures utilized in this study were similar or even greater than those produced by the spraying/sprinkler systems used in the fertigation of sugarcane crops in Brazil, ranging from 58 to 71 psi (Testezlaf, 2017). A hydraulic pump (Superagri®, 100 psi, 12 volts, 3.0 amps, flow rate of 4 L/min) whit a pressure controller, manometer, and a sprinkler nozzle used to achieve the desired pressures. A manometer was connected to this pump to measure the pressures. When the specified pressures were reached, the nematodes were passed through this system (Figure 1A) and then collected in a 10-liter plastic bucket. Next, the collected solution containing the EPNs was placed in 50 mL Falcon tubes to concentrate these organisms due to the high volume of water used in the pump. After concentration, the EPNs were quantified again and used in the following experiments. After each EPN species passed through the pressure system, it was washed with distilled water to avoid mixing different species of EPNs.

## Treatment Preparation

### Experiments (1-4) with EPNs in water

The experiments were initially conducted in water to assess the impact of increasing pressure on the infectivity of EPNs, as the combination of sugarcane substrates and pressures could potentially affect the EPNs.

### Experiment 1: Using EPNs carried in water, without sugarcane by-products

Groups of ten third instar larvae (eight to ten days old that develop on a larval development diet) of the stable fly were placed in plastic containers (7.5 x 7.5 x 4 cm) (Figure 1B) with two sheets of dry filter paper that were cut to the same size as the containers, using entomological tweezers. The IJs of *H. bacteriophora* HP88, *H. baujardi* LPP7, and *H. indica* LPP30, previously exposed to pressures of 60, 70, and 80 psi, were used. The nematodes were placed in plastic containers with the *S. calcitrans* larvae. The total volume for each treatment was four mL of distilled water, and the concentration of EPNs used was 200 IJs/larva (Leal et al., 2017). The control group had the same concentration of IJs but did not pass through the pressure system (maintained at atmospheric pressure ≈ 14.7psi). A control group without the presence of EPNs, only water, was also assessed. This assay was monitored daily for seven days. The experiment was maintained at 27±1 °C and 70%±10% RH, with six replicates.

### Experiments 2 to 4: EPNs carried in water, with sugarcane by-products

The methodology used was the same as in the previous experiment but with the prior addition of various by-products from sugarcane processing into the containers. It was used 3 g of filter cake (experiment 2), sugarcane bagasse (experiment 3), and sugarcane straw (experiment 4) per experimental unit.

### Experiments (5-8) with EPNs pressurized in vinasse

The EPNs were also carried in 50% vinasse, a concentration commonly used in the fertigation of sugarcane fields in ethanol plants in Brazil (Macedo & Carvalho, 2007).

### Experiment 5: EPNs carried in vinasse, without other sugarcane by-products

The methodology employed was similar to that described in Experiment 1. However, instead of distilled water, IJs of *H. bacteriophora* HP88, *H. baujardi* LPP7, and *H. indica* LPP30 were suspended in 50% vinasse before being exposed to pressures of 60, 70, and 80 psi and heated to 35 °C.

### Experiments 6 to 8: In vinasse, with sugarcane by-products

The methodology employed was the same as in experiment 5; however, different sugarcane by-products were previously added to the containers. Filter cake (experiment 6), sugarcane bagasse (experiment 7), and sugarcane straw (experiment 8) were used, with three grams of the substrate per experimental unit.

## Efficacy Testing and the Method Used for Assessing Mortality

Dead larvae from experiments 1 to 8 were placed in adapted White's traps (White, 1927), with adaptation, to confirm infection by EPNs by observing the adult nematodes inside the fly larvae.

## Experimental Design and Statistical Analysis

The experiments with water and vinasse were conducted under laboratory conditions (Exp. 1 to 8) using a completely randomized design. The treatments were arranged in a factorial scheme (3 x 4) + 1, which consisted of the combination of three species of EPNs (*H. bacteriophora* HP88, *H. indica* LPP30, and *H. baujardi* LPP7), four pressures (atmospheric pressure, 60, 70, and 80 psi) and an additional control treatment (without EPNs). Each treatment had six replicates. The mortality rates in treatments were corrected based on the control group (Abbott, 1925).

Initially, the data were submitted to the Shapiro-Wilk normality and Bartlett homoscedasticity tests. After observing the assumptions, the analysis of variance (ANOVA) was applied, followed by the Tukey test (*p* <0.05) to compare the means of the groups with each other. The Dunnett test (*p* <0.05) was also used to compare each group with the control group. The statistical analyses were conducted using the statistical program R version 4.0.2 (R Core Team, 2022), and the graphs were created using the Prism GraphPad 9.5.1 software.

## Results

### Experiments with EPNs in water

**Figure 2:**
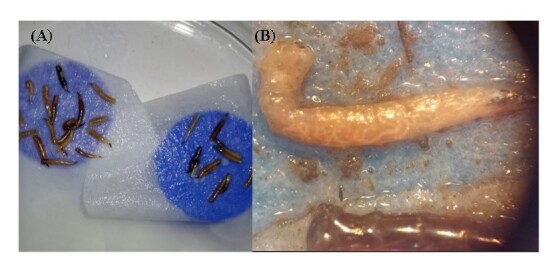
(A) White's trap (White, 1927) with dead stable fly larvae after infection with entomopathogenic nematodes. (B) Adult entomopathogenic nematodes inside the stable fly larva.

It was possible to observe the change in color of the fly larvae in White's trap (Figure 2A), becoming dark, probably due to the action of bacteria from EPNs (Dolinski, 2006). It was also possible to observe the presence of adult nematodes inside the fly larvae (Figure 2B). This color change was observed in all dead larvae, approximately 48 hours after their death.

**Figure 3:**
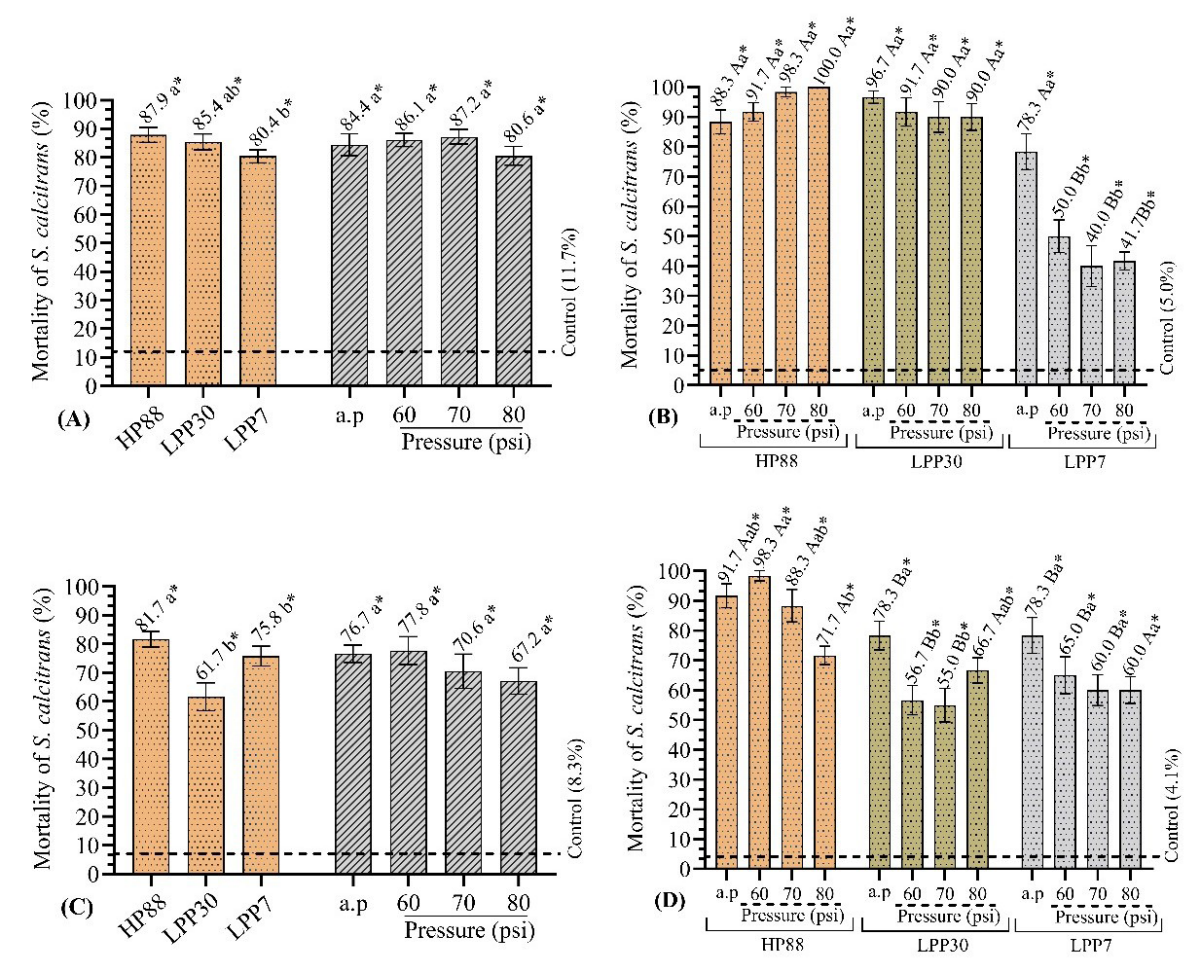
Larval mortality of *Stomoxys calcitrans* by entomopathogenic nematodes (EPNs) *Heterorhabditis bacteriophora* HP88, *H. indica* LPP30, and *H. baujardi* LPP7 subjected to different spray pressures in water. (A) EPNs in water, without sugarcane by-products. (B) Exposure of *S. calcitrans* larvae to EPNs in filter cake. (C) Exposure of *S. calcitrans* larvae to EPNs in sugarcane bagasse. (D) Exposure of *S. calcitrans* larvae to EPNs in sugarcane straw. a.p: atmospheric pressure. Means followed by the same letter are equal to each other by Tukey's test (p<0.05). * indicates statistical difference in relation to the control group by Dunnett's test (p<0.05).

In the experiment 1, there was no significant effect between EPNs and pressures. The only isolated effect observed was for nematodes, with a mean mortality of 84.6%, regardless of the pressure applied. The species *H. bacteriophora* HP88 and *H. indica* LPP30 were the most virulent to *S. calcitrans* larvae, showing mean mortality above 85%. *H. bacteriophora* HP88 (87,9%) was statistically equivalent to *H. indica* LPP30 (85,4%) but higher to *H. baujardi* LPP7 (80,4%), and although statistically similar, the pressures apparently did not affect the EPNs' virulence, as the mortality observed in all treatment groups was higher to the mortality in the control group (Figure 3A).

In the experiments with EPNs pressurized in water, applied to sugarcane by-products, the interaction between the species of EPNs and the evaluated pressures occurred in filter cake. The species *H. bacteriophora* HP88 and *H. indica* LPP30 were not affected by the pressures to which they were exposed. This indicates that the effect of these two species of EPNs on *S. calcitrans* mortality did not decay when exposed to pressures up to 80 psi. These results differ from those observed in *H. baujardi* LPP7, which exhibited decreased efficacy when exposed to 60, 70, and 80 psi (Figure 3B).

In general, *H. bacteriophora* HP88 and *H. indica* LPP30 were more efficacious than *H. baujardi* LPP7 when exposed to different pressures, indicating that the later EPN was more susceptible to the pressures used in the current study. However, under normal pressure conditions (p. atm), the three EPNs did not differ from each other.

The interaction between EPNs and pressures in sugarcane bagasse had no significant effect. Nematodes showed an isolated effect on the mortality of fly larvae, with *H. bacteriophora* HP88 being the most virulent (81.7%), whereas the other EPNs did not differ from each other. The evaluated pressures showed a mean mortality of 73.1%, with no variations among them. This indicates that the pressures did not decrease the virulence of the EPNs. Even after exposure to 60, 70, and 80 psi, the EPNs maintained their harmful effect on the fly larvae, causing a mean mortality higher than 70,0% (Figure 3C).

The tested factors interacted with one another in sugarcane straw. For *H. bacteriophora* HP88, using 80 psi resulted in a slight reduction in virulence, leading to a 71.7% larval mortality of *S. calcitrans*. Nevertheless, at pressures up to 70 psi, this species caused the mortality of fly larvae at levels higher than 88,0%. The species *H. indica* LPP30 and *H. baujardi* LPP7 showed reduced larval mortality when exposed to pressures of 60, 70, and 80 psi, although they were significantly higher than in the control group (4.1%) (Figure 3D).

Overall, *H. bacteriophora* HP88 was the most effective under all spray pressures evaluated.

### Experiments with EPNs pressurized in 50% vinasse

**Figure 4:**
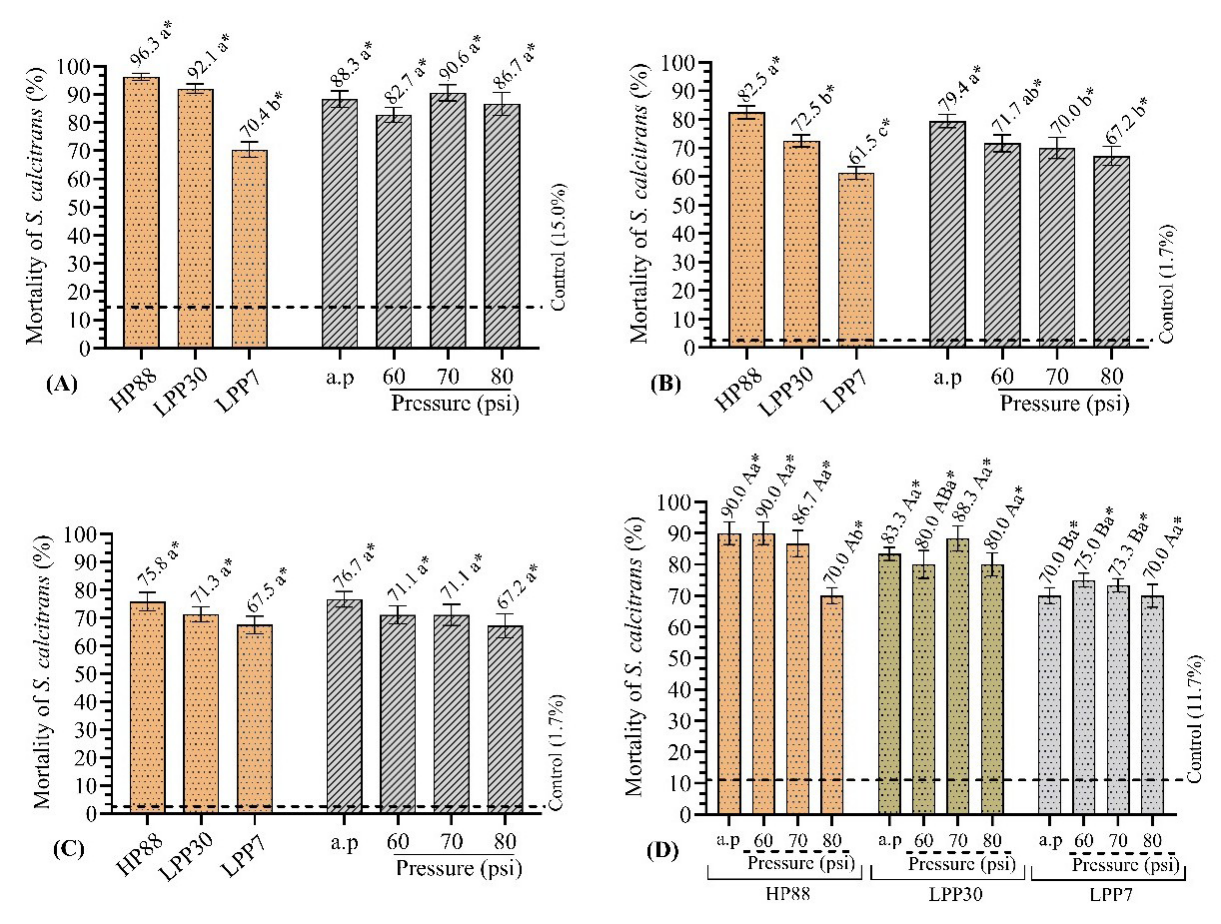
Larval mortality of *Stomoxys calcitrans* by entomopathogenic nematodes (EPNs) *Heterorhabditis bacteriophora* HP88, *H. indica* LPP30, and *H. baujardi* LPP7 subjected to different spray pressures in 50% vinasse. (A) EPNs in vinasse, without sugarcane by-products. (B) Exposure of *S. calcitrans* larvae to EPNs in filter cake. (C) Exposure of *S. calcitrans* larvae to EPNs in sugarcane bagasse. (D) Exposure of *S. calcitrans* larvae to EPNs in sugarcane straw. a.p: atmospheric pressure. Means followed by the same letter are equal to each other by Tukey's test (p<0.05). * indicates statistical difference in relation to the control group by Dunnett's test (p<0.05).

In the experiment 5, no significant variation was observed in the mean larval mortality of 87.1% due to spray pressures. This indicates that the virulence of the EPNs was not affected when exposed to different pressures. All treatments resulted in higher mean mortality compared to the control group without EPNs (15.0%). *H. bacteriophora* HP88 was statistically equal to *H. indica* LPP30, with mean mortality higher to 90%, both higher than *H. baujardi* LPP7 (70.4%) (Figure 4A).

An isolated effect was observed for the nematodes and the pressures used in the filter cake. Regardless of pressure, the nematode *H. bacteriophora* HP88 (82.5%) caused the highest mortality of stable fly larvae, followed by *H. indica* LPP30 (72.5%) and *H. baujardi* LPP7 (61.5%). Among the tested pressures, the EPNs exhibited lower virulence at 70 and 80 psi compared to atmospheric pressure (79.4%). At 60 psi, the mortality of the larvae (71.7%) showed similar behavior to the other pressures. All tested treatments had higher mean mortality than those in the control group without EPNs (1.7%) (Figure 4B).

In sugarcane bagasse, with an overall average of 71.5%, there were no variations in the mortality of *S. calcitrans* larvae when different EPNs and pressures were evaluated, although all combinations between EPN and pressure were significantly higher to the control treatment (1.7%) (Figure 4C).

The tested factors (EPNs and pressures) interacted with one another in sugarcane straw. For *H. bacteriophora* HP88, using 80 psi resulted in a reduced virulence of this EPN. However, at pressures up to 70 psi, this species caused the mortality of the fly larvae at levels higher than 86%. In the species *H. indica* LPP30, there was no decrease in larval mortality as the pressures increased because the mortality rates were statistically equal to each other for all pressures. For *H. baujardi* LPP7, the application of pressures (60, 70, and 80 psi) did not affect the performance of this nematode, causing larval mortality equal to or higher than 70% (Figure 4D). The mortality observed with *H. bacteriophora* HP88 was the same as that with *H. indica* LPP30 at all pressures. However, when comparing *H. bacteriophora* HP88 to *H. baujardi* LPP7, it was observed that *H. bacteriophora* was higher to *H. baujardi* at all pressures, except at 80 psi, where both showed statistically equal results. The EPN *H. indica* LPP30 was higher to *H. baujardi* LPP30 at atmospheric (Patm) and 70 psi pressures, being equal at 60 and 80 psi. All treated groups were higher to the control group without EPNs (11.7%).

## Discussion

EPNs have been shown to effectively control various Diptera families of economic and health importance. Cardoso et al. (2015) reported high larval mortality rates of *Aedes aegypti* Linnaeus, 1762 (Diptera: Culicidae) when exposed to *Heterorhabditis indica* LPP35, *H. indica* LPP1, and *H. baujardi* LPP31. Minas (2008) described larval mortality rates of *Ceratitis capitata* Wiedemann, 1824 (Diptera: Thephritidae) exceeding 80% when the fly larvae were exposed to *H. baujardi* LPP7. Aatif et al. (2019) used EPNs to control third instar larvae of *Bactrocera dorsalis* Hendel, 1912 (Diptera: Thephritidae). The authors observed a mortality of 69.42% when the larvae were exposed to *H. bacteriophora*.

In a study conducted by Bream et al. (2018), the action of *H. bacteriophora* on *Musca domestica* Linnaeus, 1758 (Diptera: Muscidae) larvae was investigated. The authors observed larval mortality of 100%, demonstrating the significant potential of this species of EPN in controlling housefly larvae. Mahmoud et al. (2007) utilized *Steinernema feltiae* Filipjev, 1934 to control third instar larvae of *S. calcitrans*. The researchers observed larval mortality ranging from 16.6% to 25% at a concentration of 200 EPNs/larva, proving that these EPNs were able to infect, release the symbiotic bacteria into the hemocoel of the larvae, causing sepsis and death of the fly larvae.

Leal et al. (2017) reported that the EPNs *H. bacteriophora HP88* and *H. baujardi* LPP7 caused 96.7% and 93.3% mortality, respectively, in *S. calcitrans* larvae at a concentration of 200 EPNs/larva. These authors used EPNs diluted in distilled water, as in experiments 1 to 4 in present study. In experiment 1, it was observed that the mortality caused by *H. bacteriophora* HP88 was 87.9%, whereas *H. indica* LPP30 and *H. baujardi* LPP7 had mortality rates of 85.4% and 80.4%, respectively (Figure 3).

The mortality rates presented by the EPNs by Leal et al. (2017) were higher than those found in present study. The presence of sugarcane by-products may be a factor that has impacted the performance of the EPNs in the current study. In the research conducted by Leal et al. (2017), no by-product from the sugarcane industry was added, and the EPNs were not exposed to a pressure system.

Monteiro-Sobrinho et al. (2021) demonstrated that the EPNs *H. bacteriophora* HP88 and *H. baujardi* LPP7 caused larval mortality of 91.7% and 35.0%, respectively, when the stable fly larvae were exposed to these agents for up to 48 hours. These findings are close to those found for *H. bacteriophora* HP88 in present study (87.9%) but much lower for *H. baujardi* LPP7 (80.4%) (Figure 3). This could be due to the fact that *H. baujardi* LPP7 needs more time to cause higher mortality rates, and 48 hours may be insufficient for this nematode to express all its larvicidal potential.

Monteiro-Sobrinho et al. (2016) observed that in the filter cake, in water, the nematode *H. bacteriophora* HP88 (200 EPNs/larva) was able to cause 83.3% mortality of *S. calcitrans* larvae without passing through a pressure system. This result is close to those found in the current study (experiment 2), where larval mortality ranged from 88% to 100% (Figure 3), even at the highest pressures. This indicates that regardless of the increase in pressure, the nematode *H. bacteriophora* HP88 was able to cause the death of the fly larvae at considerable levels. These findings align with Monteiro-Sobrinho et al. (2023), who reported that *H. bacteriophora* HP88 caused 83% mortality in eight-day-old *S. calcitrans* larvae in filter cake.

*H. indica* LPP30 exhibited behavior similar to that of *H. bacteriophora* HP88 in the present study, whereas *H. baujardi* LPP7 caused lower mortality rates than those described by Monteiro-Sobrinho et al. (2016), ranging from 40.0% to 78.3% (Figure 3). Its virulence decreased as the pressure increased, demonstrating that *H. baujardi* LPP7 is less virulent under these conditions than the other EPNs used.

Monteiro-Sobrinho et al. (2023) reported that *H. baujardi* LPP7 caused 80% mortality in four-day-old stable fly larvae. The study demonstrated that the younger the larva, the more susceptible it is to the EPN action, as *H. baujardi* LPP7 only caused 53% mortality of larvae that were eight days old, even without this EPN having gone through a pressure system. Even though *H. baujardi* LPP7 in filter cake does not cause larval mortality rates higher than those caused by *H. bacteriophora* HP88 and *H. indica* LPP30, these findings are still higher to those presented in other studies aimed at the biological control of stable fly larvae (Moraes et al., 2008; Alves et al., 2012).

In sugarcane bagasse, Monteiro-Sobrinho et al. (2023) reported that *H. bacteriophora* HP88 (500 EPNs/larva) caused mortality of approximately 90% of the stable fly larvae. This was more virulent than *H. baujardi* LPP7 (60%) at the same concentration of EPNs. These results correspond to those found in the current study (experiment 3), where *H. bacteriophora* HP88 (81.7%) was more virulent than *H. indica* LPP30 (61.7%) and *H. baujardi* LPP7 (75.8%), being the latter two statistically equal to one another (Figure 3). In experiment 3, the pressures had no harmful effect on the EPNS, even at the highest level.

In experiment 4, when sugarcane straw was placed in water, all nematode species tested caused larval mortality rates higher than 60%. At 60 psi, *H. bacteriophora* HP88 achieved a mortality rate of 98.3% (Figure 3). This demonstrates that EPNs can infect and kill fly larvae on the most important substrate (vinasse) in the dynamics of stable fly outbreaks associated with the sugarcane industry (Corrêa et al., 2013).

Studies involving the relationship between EPNs, vinasse, and flies are still scarce in the scientific literature. Monteiro-Sobrinho et al. (2023) showed that the EPNs *H. bacteriophora* HP88 and *H. baujardi* LPP7 were resistant to various concentrations of vinasse (50% and 100%), because the EPNs were able to survive, infect and kill fly larvae even under these conditions. They observed that *H. bacteriophora* HP88 was higher to *H. baujardi* LPP7 at both concentrations, leading to the death of over 96% of *S. calcitrans* larvae. These results align with the current study (experiment 5), which found that *H. bacteriophora* HP88 in vinasse diluted to 50% led to a 96.3% mortality of stable fly larvae, regardless of the pressure used. Meanwhile, *H. baujardi* LPP7 caused 70.4% larval mortality (Figure 4), similar to that found for the same EPN (70%) by Monteiro-Sobrinho et al. (2023). In the Monteiro-Sobrinho et al. (2023) study, it is important to note that the EPNs were not exposed to a pressure system. Yet, the results of the present study were similar to those reported by the authors mentioned above. This demonstrates that the EPNs appear to be compatible with the sprinkler/spray fertigation system used in sugarcane crops.

The experiment results with filter cake and vinasse (experiment 6) were very promising. All EPNs caused larval mortality rates higher than 60% at all pressures used. *H. bacteriophora* HP88 (82.5%) and *H. indica* LPP30 (72.5%) were the most virulent. *H. baujardi* LPP7 showed lower virulence than both but caused more than 60% mortality of stable fly larvae, regardless of the pressure (Figure 4). Hence, it is understood that in the future, control strategies for *S. calcitrans* in filter cake may also utilize EPNs, as they have proven to be efficient in this substrate, which is crucial in the dynamics of fly population explosions (Corrêa et al., 2013).

According to Corrêa et al. (2013), sugarcane bagasse is the substrate with the lowest potential for the development of immature stages of the stable fly. Once considered a problematic by-product, the sugarcane bagasse is now used for animal feed, fertilizers, raw materials for the chemical industry, and co-generation of electricity, which is one of its main applications (Silva et al., 2010). This reduces its importance in *S. calcitrans* outbreaks. However, this substrate should not be considered innocuous. The accumulation of excess bagasse in the open air creates piles, which favors the fermentation and decomposition of this material. This, in turn, attracts adults of *S. calcitrans*. As a result, it is necessary to investigate the action of EPNs on fly larvae in this by-product. In experiment 7, it was observed that *H. bacteriophora* HP88, *H. indica* LPP30, and *H. baujardi* LPP7, in bagasse, did not differ statistically from each other. They all caused a mean larval mortality of 71.5%, regardless of the pressure used (Figure 4).

Unlike sugarcane bagasse, the interaction between straw and vinasse can generate a large number of flies (Corrêa et al., 2013) but only producing fewer flies per m^2^ than the filter cake. Nonetheless, the area covered by straw with vinasse is considerably higher than the area with filter cake, making straw and vinasse the main by-products in the dynamics of population explosions of *S. calcitrans* in Brazilian sugarcane plantations (Corrêa et al., 2013).

In this scenario, EPNs have been shown to be able to resist different concentrations of vinasse (*in vitro*), causing considerable mortality against stable fly larvae (Monteiro-Sobrinho et al., 2023). In experiment 8 of the current study, it was observed that in sugarcane straw with vinasse, the EPNs were able to tolerate both 50% vinasse and the different spray pressures tested. This led to larval mortality of *S. calcitrans* at levels higher than 85% for *H. bacteriophora* HP88, 80% for *H indica* LPP30, and 70% for *H. baujardi* LPP7 (Figure 4). Therefore, EPNs were capable of infecting and killing stable fly larvae in the most important by-products associated with the sugarcane industry (Corrêa et al., 2013) at the pressures commonly used in the fertigation of sugarcane crops (Testezlaf, 2017).

Lara et al. (2008a) exposed *H. baujardi* LPP7 to a micro-sprinkler irrigation system with 20-35 psi. They observed that in addition to the EPN resisting the pressures used, these organisms could cause 85% larval mortality in *Galleria mellonella* (Lepidoptera: Pyralidae). In the present study, the same species of EPN used by Lara et al. (2008a) was able to cause mortality rates higher than 60% in practically all biological tests with *S. calcitrans*. The highest mortality observed in *G. mellonella* larvae compared to *S. calcitrans* larvae may be attributed to the fact that *G. mellonella* is significantly more susceptible to the action of EPNs than the stable fly. This susceptibility is evident since it is usually used in the *in vivo* maintenance of EPN colonies (Lindegren et al., 1993). Furthermore, the pressures employed in present study are much higher than those used by Lara et al. (2008a). However, Lara et al. (2008b) found that *H. baujardi* LPP7 could withstand pressures of up to 340 psi, which is much higher than those used in the current study.

Fife et al. (2003) reported that *H. bacteriophora* can tolerate pressures up to 290 psi. Nevertheless, these authors noted that the viability of *H. bacteriophora* decreased as the pressure increased to limits beyond 180 psi. According to Grewal (2002), EPNs generally should not be exposed to pressures higher than 300 psi. Therefore, it is probable that EPNs exposed to such high pressures would not cause considerable mortality rates in *S. calcitrans,* especially *H. baujardi LPP7*, which was found to be sensitive to the increase in pressure in some biological tests in the current study.

## Conclusion

The EPNs *H. bacteriophora* HP88, *H. indica* LPP30 and *H. baujardi* LPP7, exposed to spray pressures in laboratory, maintain their ability to infect and kill stable fly larvae in sugarcane byproducts. EPN’s efficacy is influenced by the spray pressure and carrier, as well as by the organic substrate where stable fly larvae are developing.

## Data Availability

Data are not available in virtual databases.

## References

[B001] Aatif HM, Hanif MS, Ferhan M, Raheel M, Shakeel Q, Ashraf W (2019). Assessment of the entomopathogenic nematodes against maggots and pupae of the oriental fruit fly, *Bactrocera dorsalis* (Hendel) (Diptera: Tephritidae), under laboratory conditions. Egypt J Biol Pest Control.

[B002] Abbott WS (1925). A method of computing the effectiveness of on an insecticide. J Econ Entomol.

[B003] Alves SB (1998). Controle microbiano de insetos..

[B004] Alves PS, Moraes APR, Salles CMC, Bittencourt VREP, Bittencourt AJ (2012). Lecanicillium lecanii no controle de estágios imaturos de Stomoxys calcitrans.. Braz J Vet Med.

[B005] Bream AS, Fouda MA, Ibrahim E, Shehata IE, Ragab SH (2018). Evaluation of four entomopathogenic nematodes as biological control agents against the housefly, *Musca domestica* L. (Diptera: muscidae). Egypt Acad J Biolog Sci.

[B006] Barros ATM, Koller WW, Catto JB, Soares CO (2010). Surtos por *Stomoxys calcitrans* em gado de corte no Mato Grosso do Sul. Pesq Vet Bras.

[B007] Barros ATM, Rodrigues VD, Cançado PHD, Domingues LN (2019). Resistance of the stable fly, *Stomoxys calcitrans* (Diptera: Muscidae), to cypermethrin in outbreak areas in Midwestern Brazil. Rev Bras Parasitol Vet.

[B008] Bittencourt AJ (2012). Avaliação de surtos e medidas de controle ambiental de *Stomoxys calcitrans* (Diptera: Muscidae) na Região Sudeste do Brasil. Rev Bras Med Vet.

[B009] Cardoso DO, Gomes VM, Dolinski C, Souza RM (2015). Potential of entomopathogenic nematodes as biocontrol agents of immature stages of *Aedes aegypti.*. Nematoda.

[B010] Corrêa EC, Ribas ACA, Campos J, Barros ATM (2013). Abundância de *Stomoxys calcitrans* (Diptera: Muscidae) em diferentes subprodutos canavieiros. Pesq Vet Bras.

[B011] Dolinski C, Oliveira EC, Monnerat RG (2006). Fundamentos para regulação de semioquímicos inimigos naturais e agentes microbiológicos de controle de pragas..

[B012] Dolinski C, Moino A (2006). Utilização de nematóides entomopatogênicos nativos ou exóticos: o perigo das introduções. Nematol Bras.

[B013] Dominghetti TFS, Barros ATM, Soares CO, Cançado PHD (2015). *Stomoxys calcitrans* (Diptera: Muscidae) outbreaks: current situation and future outlook with emphasis on Brazil. Rev Bras Parasitol Vet.

[B014] Fife JP, Derksen RC, Ozkan HE, Grewal PS (2003). Effects of pressure differentials on the viability and infectivity of entomopathogenic nematodes. Biol Control.

[B015] Grewal PS, Gaugler R (2002). Entomopathogenic nematology..

[B016] Grisi L, Leite RC, Martins JR, Barros ATM, Andreotti R, Cançado PHD (2014). Reassessment of the potential economic impact of cattle parasites in Brazil. Rev Bras Parasitol Vet.

[B017] Koppenhöfer AM, Grewal PS, Grewal PS, Ehlers RU, Shapiro-Ilan DI (2005). Nematodes as biological control agents..

[B018] Lara JC, Dolinski C, Sousa EF, Daher RF (2008). Effect of mini-sprinkler irrigation system on *Heterorhabditis baujardi* LPP7 (Nematoda: Heterorhabditidae) infective juvenile. Sci Agric.

[B019] Lara JC, Dolinski C, Sousa EF (2008). Viability, infectivity, and search capability of *Heterorhabditis baujardi* LPP7 (Nematoda: Heterorhabditidae) under different pressure and temperature conditions. Nematol Bras.

[B020] Leal LCSR, Monteiro CMO, Mendonça AE, Bittencourt VREP, Bittencourt AJ (2017). Potential of entomopathogenic nematodes of the genus *Heterorhabditis* for the control of *Stomoxys calcitrans* (Diptera: muscidae). Rev Bras Parasitol Vet.

[B021] Lindegren JE, Valero KA, Mackey BE (1993). Simple *in vivo* production and storage methods for *Steinernema carpocapsae* infective juveniles. J Nematol.

[B022] Macedo IC, Carvalho EP (2007). A energia da cana-de-açúcar: Doze estudos sobre a agroindústria da cana-de-açúcar no Brasil e a sua sustentabilidade..

[B023] Mahmoud MF, Mandour NS, Pomazkov IY (2007). Efficacy of the entomopathogenic nematode *Steinernema feltiae* cross n 33 against larvae and pupae of four fly species in the laboratory. Nematol Mediterr.

[B024] Mello RP (1989). Estudo de alguns aspectos do desenvolvimento biológico e do comportamento, em laboratório, de Stomoxys calcitrans, (Linnaeus, 1758) (Diptera: Muscidae).

[B025] Minas RS (2008). Avaliação do potencial dos nematóides entomopatogênicos como agentes de controle biológico da moscas-das-frutas, Ceratitis capitata (Wiedemann) (Diptera: Tephritidae) na cultura da goiaba (Psidium guajava).

[B026] Monteiro CMO, Matos RS, Araújo LX, Campos R, Bittencourt VREP, Dolinski C (2014). Entomopathogenic nematodes in insect cadaver formulations for the control of *Rhipicephalus microplus* (Acari: ixodidae). Vet Parasitol.

[B027] Monteiro-Sobrinho AC, Costa ILA, Souza GC, Leal LCSR, Monteiro JLL, Chambarelli MCMC (2021). Infection and reinfection of *Stomoxys calcitrans* larvae (Diptera: Muscidae) by entomopathogenic nematodes in different times of exposure. Rev Bras Parasitol Vet.

[B028] Monteiro-Sobrinho AC, Leal LCSR, Monteiro JLL, Chambarelli MCMC, Bittencourt AJ (2023). Evaluation *in vitro* of the virulence of two entomopathogenic heterorhabditid nematodes in the control of *Stomoxys calcitrans* (Diptera: Muscidae) larvae in byproducts of the sugar and alcohol industry. Rev Bras Parasitol Vet.

[B029] Monteiro-Sobrinho AC, Mendes COF, Leal LCSR, Bittencourt AJ (2016). Virulência de *Heterorhabditis bacteriophora* cepa HP88 (Rhabditida: Heterorhabtidae) sobre larvas de *Stomoxys calcitrans* (Diptera: Muscidae) em dieta de torta de filtro. Rev Bras Med Vet.

[B030] Moraes APR (2007). Stomoxys calcitrans: estabelecimento de colônia e efeito de Metarhizium anisopliae sobre seus estágios imaturos.

[B031] Moraes APR, Angelo IC, Fernandes EKK, Bittencourt VREP, Bittencourt AJ (2008). Virulence of *Metarhizium anisopliae* to eggs and immature stages of *Stomoxys calcitrans.*. Ann N Y Acad Sci.

[B032] Mullens BA, Meyer JA (1987). Seasonal abundance of stable flies (Diptera: Muscidae) on california dairies. J Econ Entomol.

[B033] R Core Team (2022). R: A language and environment for statistical computing.

[B034] Silva VS, Garcia CA, Silva CM (2010). O destino do bagaço da cana-de-açúcar: um estudo a partir das agroindústrias. Rev Agronegócio Meio Ambient.

[B035] Souza TF, Cançado PHD, Barros ATM (2021). Attractivity of vinasse spraying to stable flies, *Stomoxys calcitrans* (Diptera: Muscidae), in a sugarcane area. Pesq Vet Bras.

[B036] Taylor DB, Moon RD, Mark DR (2012). Economic impact of stable flies (Diptera: Muscidae) on dairy and beef cattle production. J Med Entomol.

[B037] Taylor DB, Szalanski AL, Adams BJ, Peterson RD (1998). Susceptibility of house fly (Diptera: Muscidae) larvae to entomopathogenic nematodes (Rhabditida: Heterorhabditidae, Steinernematidae). Environ Entomol.

[B038] Testezlaf R (2017). Irrigação: métodos, sistemas e aplicações..

[B039] White GF (1927). A method for obtaining infective nematode larvae from cultures. Science.

